# Blackcurrant (*R. nigrum*) Pomace Extract-Loaded Hydrogels: A Sustainable Alginate–Inulin Matrix for Polyphenol Delivery

**DOI:** 10.3390/gels12100929

**Published:** 2026-10-15

**Authors:** Diāna Brūvere, Ance Bārzdiņa, Renārs Broks, Rudīte Koka, Renāte Teterovska

**Affiliations:** 1 Department of Applied Pharmacy, Faculty of Pharmacy, Rīga Stradiņš University, LV-1007 Riga, Latvia; 2 Baltic Biomaterials Centre of Excellence, Riga Technical University, P. Valdena Str. 3, LV-1048 Riga, Latvia; 3 Department of Biology and Microbiology, Rīga Stradiņš University, LV-1007 Riga, Latviarudite.koka@rsu.lv (R.K.)

**Keywords:** *Ribes nigrum*, polysaccharide-based gel, hydrogel, hydrogel drug delivery, antibacterial activity, pomace, polyphenol, valorization

## Abstract

Blackcurrant (*Ribes nigrum*) is rich in polyphenols and is known for its antioxidant and anti-inflammatory properties, yet its processing byproducts (pomace) remain underutilized. This study aimed to develop and characterize *R. nigrum* pomace extract-loaded alginate–inulin hydrogel for potential topical application, thus promoting the valorization of *R. nigrum* pomace extract. Comparative extract analysis demonstrated that the pomace extract contained the highest levels of total polyphenols (71.0 mg GAE/g dried extract) and tannins (21.8%) and exhibited the most promising antibacterial activity (MIC 3.1–6.3 mg/mL), although its antiradical activity was relatively lower than that of juice and berry extracts. Furthermore, the obtained hydrogels were structurally stable and provided a gradual release of polyphenols at pH 5.5, with 80% released within 24 h. Incorporation of *R. nigrum* pomace extract increased the swelling of the hydrogels and improved viscoelastic recovery. Overall, the findings highlight the potential of *R. nigrum* pomace as a sustainable bioactive component in alginate–inulin hydrogel systems for potential topical applications in wound care.

## 1. Introduction

*Ribes nigrum L*., commonly known as blackcurrant, is a nutrient-rich berry from the *Grossulariaceae* family. It is widely recognized for its high content of vitamin C and polyphenolic compounds, mostly anthocyanins [1]. Both the berries and leaves of *R. nigrum* have been valued in traditional medicine for their antioxidant and anti-inflammatory properties [2,3]. In the last five years, scientific interest in *R. nigrum* has increased (with 775 scientific articles on PubMed as of 10 February 2026; keywords: blackcurrant; *Ribes nigrum*) due to its potential therapeutic and cosmetic applications, attributed to bioactive phytochemicals that can modulate oxidative stress and inflammatory responses [4,5]. While *R. nigrum* berries are commonly processed into juice, porridge, jams, and marmalade, the resulting berry pomace is often discarded as industrial waste [6]. However, these byproducts are a valuable and underutilized source of bioactive compounds.

The valorization of agricultural and food industry byproducts supports more sustainable and resource-efficient processing and contributes to zero-waste processing models. Using *R. nigrum* berry-derived products could not only reduce environmental impact but also create new opportunities for farmers and manufacturers [7]. This pomace, when processed into standardized extracts, may find applications in the pharmaceutical and cosmetic industries due to its bioactive properties. In this context, hydrogel-based skin care formulations represent an advanced form of topical treatment, as their three-dimensional polymeric structure can maintain moisture balance, regulate sebum, and enable localized delivery of bioactive agents [8,9].

Among natural hydrogel-forming polymers, alginate and inulin are highly promising for skin application due to their biocompatibility and multifunctional properties. Alginate is valued for its gel-forming ability, high exudate absorption capacity, and hemostatic properties, helping to maintain a moist environment [10]. Inulin forms hydrogels with high water absorption and controlled release properties, and a biodegradable matrix [11]. Together, the alginate–inulin system provides a protective and physiologically favorable microenvironment, generally does not provoke immune responses, and its therapeutic potential can be further enhanced by incorporating *R. nigrum* pomace ethanol extract, whose phytochemical properties may further support topical delivery [3,12].

Although alginate–inulin systems have been investigated as carriers for berry-derived bioactive extracts, including *Clindemia rubra* and *Aronia melanocarpa* extracts [13], these studies primarily addressed encapsulation and gastrointestinal or general drug-delivery applications rather than topically used hydrogels for potential wound care. Previously, *R. nigrum* extracts have been incorporated into other hydrogel matrices, including gelatin-oxidized pullulan and alginate-based systems [14]. Thus, the use of an extract derived specifically from *R. nigrum* berry pomace in an alginate–inulin hydrogel designed for potential antioxidant and antibacterial topical applications represents a distinct and novel formulation approach.

This study aimed to develop and characterize an *R. nigrum* pomace extract-loaded alginate–inulin hydrogel for potential topical polyphenol delivery and thus promote the valorization of *R. nigrum* pomace extract. To achieve this aim, the study assessed the polyphenolic profile, antibacterial activity, and antioxidant activity of the ethanol extract of *R. nigrum* pomace and compared it with the ethanol extracts of *R. nigrum* berries and juice. Furthermore, the study developed an alginate–inulin hydrogel containing *R. nigrum* pomace extract and determined its physicochemical and structural properties. This approach may promote the sustainable use of berry-processing byproducts and support the development of functional biomedical hydrogel formulations.

## 2. Results and Discussion

### 2.1. Chemical Characterization of Extracts

#### 2.1.1. Determination of the Tannin Content in the Extract

**Figure 1:**
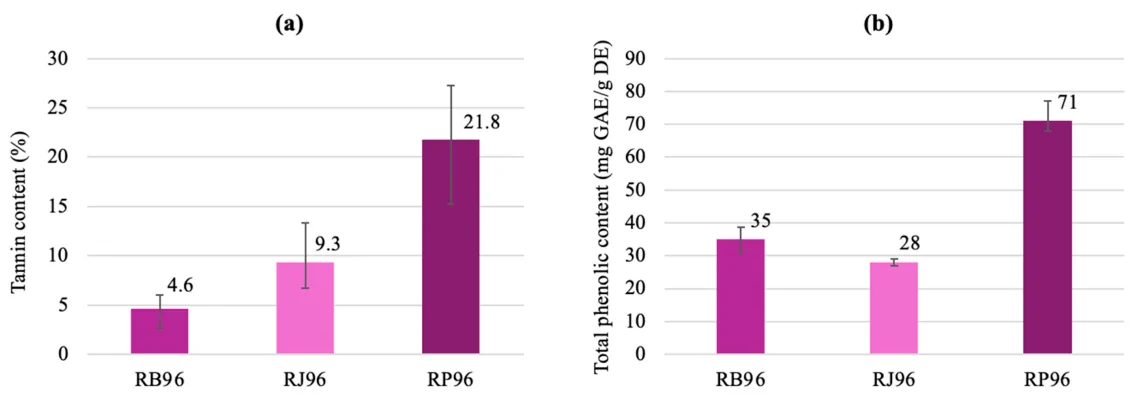
(**a**) Tannin content in *R. nigrum* extract; (**b**) total phenolic content in *R. nigrum* extract.

The comparative analysis of tannin concentrations revealed substantial differences, depending on the type of plant material (Figure 1a). All values are expressed as mean ± SD (*n* = 3). The SD was calculated separately for each sample. RP96 yielded the highest tannin content (21.8%), almost twice as high as that of any tested *R. nigrum* berry extract. This observation can be explained by the anatomical distribution of condensed tannins, which predominantly accumulate in skins and seeds rather than in the pulp, making pomace particularly rich in these compounds [15]. RB96 and RJ96 yielded significantly lower tannin levels (4.6% and 9.3%, respectively). This indicates that soluble tannins remain present in the liquid fraction but at lower concentrations compared to solid residues.

#### 2.1.2. Total Phenolic Content

Analysis of TPC, expressed as gallic acid equivalents, is shown in Figure 1b. Once again, RP96 exhibited the highest phenolic content (71.0 mg GAE/g dried extract), indicating that a substantial proportion of phenolic constituents is retained in the solid residue after juice processing. Results confirm its value as a phenolic-rich byproduct [16].

#### 2.1.3. Determination of Polyphenol Profiles with HPLC-UV Assay

**Figure 2:**
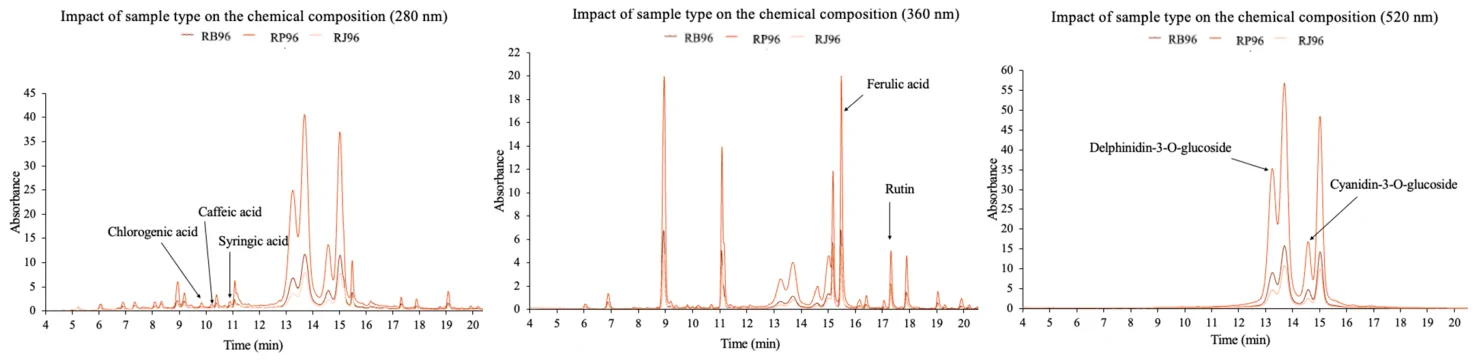
Impact of sample type on chemical composition.

HPLC-UV analysis revealed almost identical polyphenol profiles between pomace, whole berry, and juice extracts (Figure 2). The primary difference between extract types lies in the concentration of key compound classes such as anthocyanins. Anthocyanins have two absorption maxima—one in the visible light range (510–530 nm) and one in the ultraviolet light range (270–280 nm) [17,18]. Therefore, they can be seen on chromatograms at both 280 nm and 520 nm, hindering the detection of compounds with the same retention time. Indicated by peak intensity, the highest concentration of anthocyanins was detected in RP96, while the lowest was in RJ96. Two anthocyanins—delphinidin-3-O-glucoside and cyanidin-3-O-glucoside—were identified. Based on the literature data with similar absorption patterns, the other two anthocyanin peaks detected could be delphinidin-3-O-rutinoside and cyanidin-3-O-rutinoside [19,20]. Based on the chromatographic profiles, the highest concentration of anthocyanins was extracted from RP96 (Figure 2). Besides anthocyanins, other polyphenols such as ferulic acid, p-coumaric acid, chlorogenic acid, caffeic acid, and rutin were successfully identified in all tested extracts. Among the phenolic acids, ferulic acid had the most intense peak. To inspect the polyphenolic composition in more detail, HPLC-MS/MS analysis could be used in the future.

### 2.2. Biological Activity of R. nigrum Extracts

Reactive oxygen species (ROS) induce damage to the wound microenvironment [21]. In evaluating the potential of *R. nigrum* extracts for topical delivery, determining their antiradical (DPPH) and antibacterial activities is essential. Quantitatively, the DPPH test indicates the antioxidative capacity of the extracts and the association of phenolic compounds [22,23]. Bacterial infections delay wound healing; therefore, antibacterial activity of the extracts was assessed.

#### 2.2.1. Antiradical Activity (DPPH Assay)

**Figure 3:**
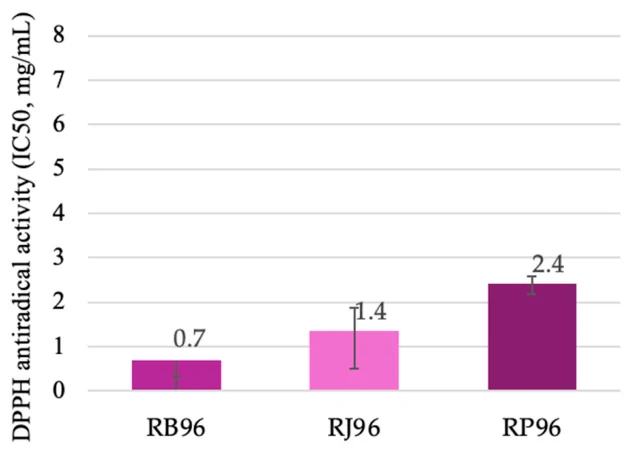
Antiradical activity of *R. nigrum* extracts.

Evaluating DPPH antiradical activity showed clear differences between *R. nigrum L.* extracts obtained with different ethanol concentrations (Figure 3). All values are expressed as mean ± SD (*n* = 3). The SD was calculated separately for each sample. The IC50 value, which represents the extract concentration required to neutralize 50% of DPPH free radicals, ranged from 0.7 mg/mL to 2.4 mg/mL, indicating antioxidant potential. The most effective antioxidant activity (lowest IC50 value) was observed for the RB96 (0.7 mg/mL). The RJ96 also exhibited high activity (1.4 mg/mL). RP96, despite its highest tannin and phenolic levels, displayed only moderate activity (2.4 mg/mL), suggesting that not all phenolic compounds contribute equally to antiradical effects. Notably, total polyphenol content does not always correlate directly with DPPH-scavenging activity, as individual compounds vary significantly in their radical-scavenging efficiency [24,25]. Furthermore, the DPPH assay relies on specific hydrogen atom and/or electron transfer mechanisms under defined conditions and thus may not capture the full spectrum of antioxidant mechanisms operating in complex plant matrices [26].

#### 2.2.2. Antibacterial Activity of the Extracts

The minimum inhibitory concentration (MIC) and minimum bactericidal concentration (MBC) values of tested extracts against *E. coli* and *S. aureus* are shown in Table 1. All values are expressed as mean (*n* = 3). Based on the results, *R. nigrum* extracts showed moderate or low antibacterial activity against both bacteria [27]. The higher polyphenolic and tannin content in the pomace extract may contribute to its antibacterial activity [28]. However, based on the present data, the observed antibacterial effect cannot be attributed to a single compound. The higher concentration of phenolic acids, flavonoids, and anthocyanins detected in the pomace extract suggests that its antibacterial activity may result from the combined action of several phenolic constituents. The weakest antibacterial activity against *E. coli* was observed in the RJ96 extract (MIC 12.5 mg/mL, MBC 25 mg/mL). The strongest antibacterial activity among all tested extracts was shown by RP96 extract, which had the lowest MIC (3.1 mg/mL) and MBC (6.3 mg/mL). The MBC/MIC ratio is presented in Table 1. All extracts showed bactericidal activity against both bacteria (MBC/MIC < 4) [29]. Overall, *S. aureus* proved more sensitive to the tested extracts than *E. coli,* as reflected by a lower MBC/MIC ratio. This aligns with previous studies indicating a higher susceptibility of *S. aureus* [30]. Literature reports on *E. coli* susceptibility to *R. nigrum* preparations are inconsistent. Some studies report weak or no inhibition, especially for juice-based samples, while others show moderate antibacterial effects [30,31,32]. These variations likely result from differences in *E. coli* strains, extract type, extraction methods, and test conditions. Generally, the antibacterial activity of *R. nigrum* extracts is compatible with other unpurified plant extracts but remains insufficient compared to drugs for optimal antibacterial efficacy [33]. Additionally, comparison with individual purified natural compounds is complex, as the antibacterial activity of plant extracts may result from additive or synergistic interactions among several constituents and therefore cannot be directly represented by individual compounds [34]. This highlights the need for further studies to assess potential synergistic effects between natural compounds themselves and with conventional antibacterial agents.

### 2.3. Structural Characterization of RP96-Loaded Alginate–Inulin Hydrogels

#### 2.3.1. FTIR-ATR Spectral Fingerprint

**Figure 4:**
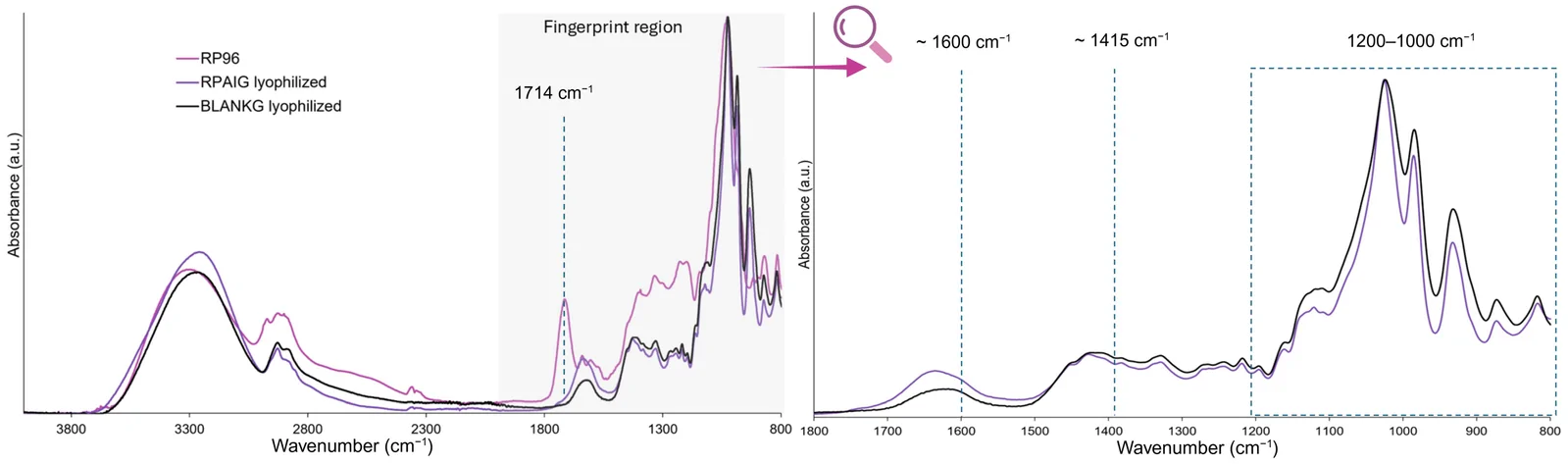
FTIR-ATR spectral fingerprint of RP96 extract and alginate–inulin hydrogels. Full spectra of RP96 extract, BLANKG, and RPAIG in the 3800–600 cm^−1^ range. Enlarged fingerprint region of BLANKG and RPAIG in the 1800–800 cm^−1^ range. Spectra were normalized before comparison.

FTIR-ATR spectra of RP96 extract, BLANKG, and RPAIG are shown in Figure 4. The full spectra (Figure 4) demonstrate clear differences between the RP96 extract and the alginate–inulin hydrogel matrix, while the RPAIG spectrum largely follows the spectral profile of BLANKG. This indicates that incorporation of RP96 did not substantially alter the main polysaccharide matrix of the hydrogel.

The enlarged fingerprint region (1800–800 cm^−1^, Figure 4) was used to compare BLANKG and RPAIG in more detail. The main bands associated with the alginate–inulin matrix were retained after RP96 incorporation, especially the carboxylate-related bands around 1600 and 1415 cm^−1^ and the polysaccharide C–O/C–O–C region at approximately 1200–1000 cm^−1^. The retention of the carboxylate-related bands in RPAIG suggests that the Ca^2+^-crosslinked alginate network was largely preserved after RP96 incorporation. The characteristic RP96 band around 1714 cm^−1^, attributed to C=O stretching of phenolic acid- or flavonoid-related structures, was not clearly resolved in RPAIG, due to the low extract concentration and overlap with the broad hydrogel matrix bands.

Minor differences in band shape within the 1600–1000 cm^−1^ region may reflect overlapping contributions from RP96 phenolic constituents and the alginate–inulin network. These changes suggest possible weak non-covalent interactions, such as hydrogen bonding, and possible interactions involving carboxylate groups within the Ca^2+^-crosslinked alginate matrix.

However, no new, clearly resolved bands or major spectral shifts were observed. Therefore, the FTIR-ATR data support the incorporation of RP96 into the alginate–inulin hydrogel system and indicate the preservation of the main hydrogel matrix, with no evidence of substantial chemical modification or structural disruption.

#### 2.3.2. X-Ray Diffraction Profile

**Figure 5:**
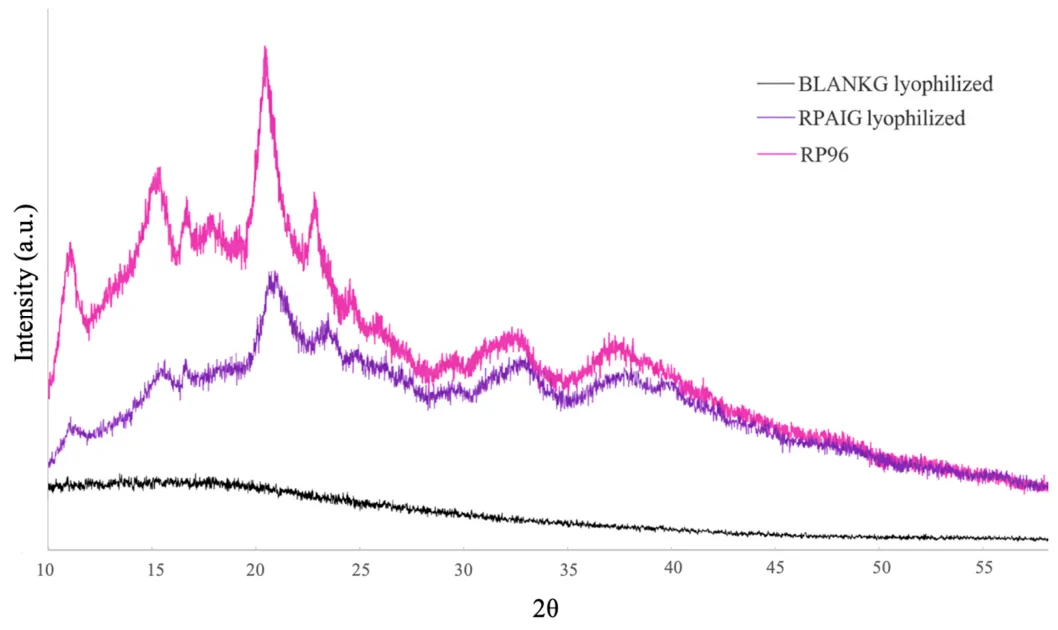
Comparison of crystallinity of the RP96 extract and resulting hydrogels with the X-ray diffraction test.

XRD patterns of the RP96 and the resulting hydrogels are shown in Figure 5. The dried RP96 extract shows diffraction peaks between 12–24° (2θ), suggesting partially ordered or microcrystalline small molecules in the plant matrix. Similar patterns are seen in other dried botanicals, where residual sugars and small organic compounds (such as phenolic acids) produce weak but noticeable XRD reflections in biomass extracts [35]. In contrast, the blank alginate–inulin hydrogel exhibits a predominantly amorphous diffraction profile characterized by a broad halo and absence of sharp reflections, confirming the disordered polymer network formed after gelation and Ca^2+^ crosslinking.

After incorporating RP96 into the matrix, the diffraction pattern remains amorphous, while the extract-related peaks become attenuated and broadened. This indicates effective dispersion of the extract components within the polymer network without inducing crystallization of the alginate–inulin structure.

### 2.4. Rheological Behavior of Alginate–Inulin Hydrogels

Rheological measurements were performed to determine the viscoelastic properties of the material, characterizing its stiffness, flow behavior, and structural stability under mechanical loading. These measurements enable assessment of the hydrogel’s mechanical strength, deformation limits, and suitability for its intended application.

#### 2.4.1. Viscoelastic Stability Under Oscillatory Deformation

**Figure 6:**
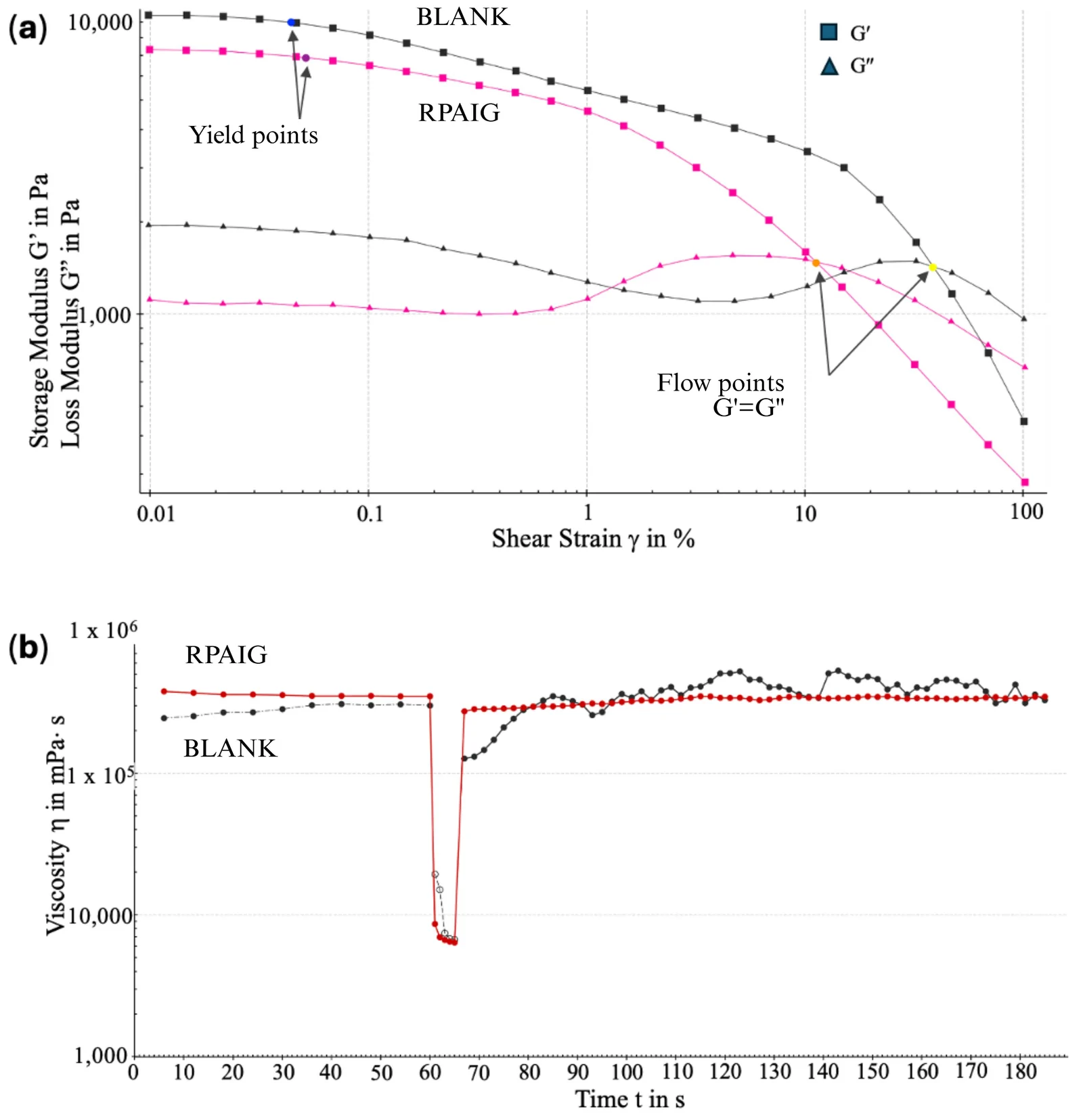
(**a**) Viscoelastic properties of BLANK and RPAIG; (**b**) comparison of viscosity recovery of BLANKG and RPAIG in the 3iTT test.

The ASW test compared hydrogels in terms of stiffness, stability, and the onset of structural breakdown, as shown in Figure 6a. Both the RPAIG and the BLANKG exhibited typical gel-like behavior, with the storage modulus (G′) exceeding the loss modulus (G″). The BLANKG showed a higher initial G′ (10.1 kPa) than the RPAIG (8.9 kPa), indicating a stiffer structure. With increasing strain, both hydrogels showed a gradual decrease in G′, reflecting the onset of irreversible structural deformation. This decrease was slower for the BLANKG, suggesting a more durable and stable structure. Despite this, the RPAIG showed a slightly larger linear viscoelastic region (γ ≈ 0.051% compared to 0.045%), suggesting a more elastic but less rigid structure that can accommodate slightly larger small deformations before the onset of irreversible structural changes. The flow point (G′ = G″) occurred at γ ≈ 11% for RPAIG and at γ ≈ 39% for BLANKG.

#### 2.4.2. Thixotropic Properties of Alginate–Inulin Hydrogels

The thixotropic properties of alginate–inulin hydrogels, with and without RP96, were assessed using a 3ITT (Figure 6b). In the first interval, both hydrogels exhibited high viscosity characteristics of a structured hydrocolloid system. However, the RPAIG showed a significantly higher initial viscosity (~3.0 × 10^5^ mPa·s) compared to the BLANKG matrix (~2.1 × 10^5^ mPa·s), suggesting that *R. nigrum* polyphenols enhance the interactions within the hydrogel structure, promoting a more interconnected and dynamically stable viscoelastic structure. In the second interval, both hydrogels experienced a rapid decrease in viscosity. Significant differences were found in the third interval. The RPAIG stabilizes faster compared to the BLANKG, which wobbles and is unable to regain balance. These results demonstrate that the presence of berry polyphenols not only strengthens the initial mechanical structure of the hydrogel but also improves its ability to form a stable structure with higher resistance to deformation.

#### 2.4.3. Qualitative Assessment of Macroscopic Self-Healing Behavior Under Unstimulated Conditions

**Figure 7:**
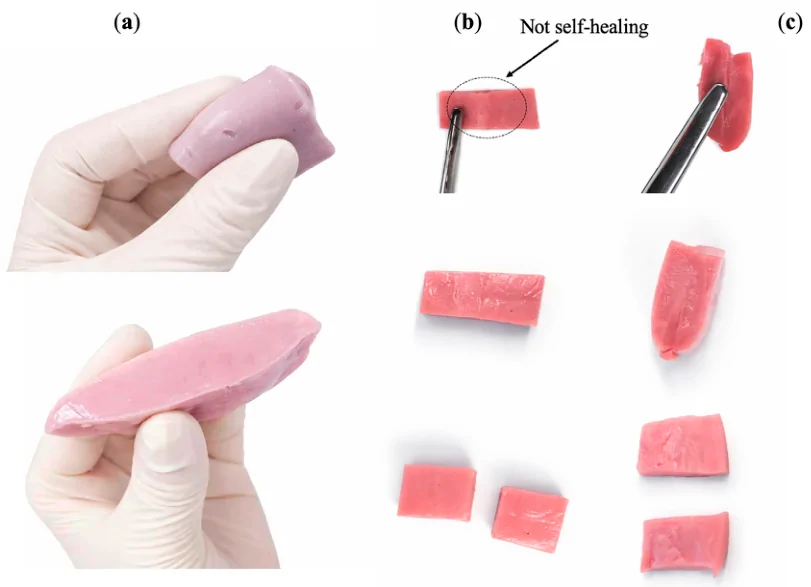
Qualitative assessment of macroscopic self-healing hydrogel tests after 1 h and 24 h. (**a**) Hydrogel flexibility; (**b**) 1 h test; (**c**) 24 h test.

The hydrogel recovery was assessed visually to compare with rheological analysis (Figure 6a). The rheological results indicated structural recovery; however, in the qualitative self-healing hydrogel test without external stimulus, the BLANKG and RPAIG showed negative results in the 1 h and 24 h tests (Figure 7). Alginate–inulin hydrogels contain a large amount of water [36]. When two smooth hydrogel surfaces come into contact, a very thin water film or liquid bridge forms between them. This water film creates surface tension [37]. The water molecules try to reduce the free surface, creating an attractive force that pulls the two hydrogel halves together. This force is the capillary force. Since the cut surfaces were smooth, this capillary force is strong enough to hold the two halves together when they are lifted, because the force acts perpendicular to the surface. When each half is pulled in its own direction (shear stress, surface separation stress), this relatively weak water bridge is easily broken. Thus, the observed interaction between the hydrogel surfaces cannot be attributed exclusively to self-healing, as interfacial adhesion and capillary bridging may also contribute. These observations suggest that the system using RP96 did not enhance the macroscopic self-healing properties of the alginate–inulin hydrogel under the unstimulated conditions tested. However, the qualitative nature of the test does not allow the complete absence of self-healing capability to be conclusively established, as the test was qualitative and did not quantify the self-healing of mechanical properties.

Although the RP96 improved rheological recovery, it did not enhance macroscopic self-healing under the conditions tested. Since qualitative tests cannot distinguish self-healing from interfacial adhesion or capillary bridging, further quantitative mechanical testing is required. Future studies could focus on further improving hydrogel properties, particularly the balance between mechanical stability, structural recovery, and macroscopic self-healing.

### 2.5. Hydrogel Swelling

**Figure 8:**
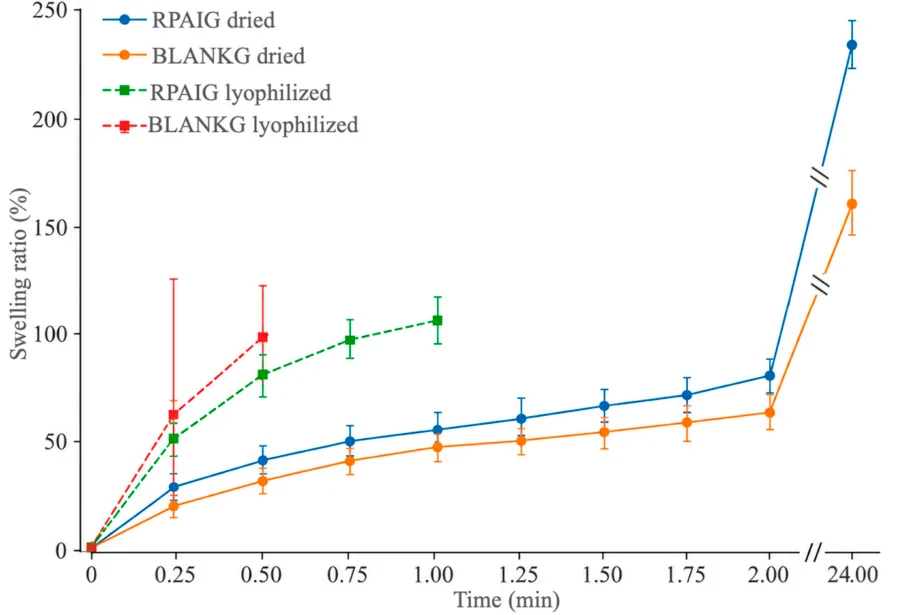
Effect of RP96 on swelling of room temperature dried and lyophilized hydrogels.

Hydrogel swelling at pH 5.5 showed marked differences between the compositions (RPAIG vs. BLANKG) and between dried and lyophilized matrices (Figure 8). Dried RPAIG in the 0–120 min interval: after 120 min, the swelling of dried RPAIG reaches 79%, while dried BLANKG reaches only 64%, but after 24 h, these values are 240% and 161%, respectively. These results indicate that *R. nigrum* polyphenols significantly increase the hydrophilicity and water absorption capacity of the matrix, which is consistent with the rheological results (lower G′). A similar observation, where polyphenols improve hydrogel swelling, has been described in the literature by Josian Moreira Mar using Amazonian berry extracts and alginate–inulin systems [13]. The opposite situation is observed in the lyophilized hydrogels. Lyophilized BLANKG showed very rapid swelling, more than 100% within 0.25 h, and further structural collapse within 0.5 h, indicating high porosity and low mechanical coherence after lyophilization. Lyophilized RPAIG, although swollen similarly, maintained its integrity for longer. The sample lost mechanical stability within one hour. *R. nigrum* polyphenols form additional interactions with the alginate–inulin porous system, which temporarily increases mechanical integrity. This is also confirmed in the 3ITT test, where RPAIG showed a higher initial viscosity and more stable recovery.

The increased swelling observed after RP96 incorporation may be related to weak non-covalent interactions between phenolic constituents and the alginate–inulin matrix, particularly hydrogen bonding and possible interactions involving alginate carboxylate groups. Such interactions may modify the effective network density and swelling. Our FTIR-ATR results showed minor changes in the 1600–1000 cm^−1^ region, consistent with possible interactions between RP96 constituents and the polymer matrix, while the main carboxylate and polysaccharide bands were retained, indicating preservation of the Ca^2+^-crosslinked alginate network without substantial structural disruption. The increased swelling is likely associated with changes in polymer–polyphenol interactions and hydration of the network rather than disruption of the Ca^2+^-crosslinked structure.

Overall, the pH 5.5 conditions ensure that the calcium alginate–inulin matrix retains its cross-linking and hydrogel structure [38]. Therefore, the observed differences between the samples mainly reflect the effects of matrix composition and drying history, rather than the chemical breakdown of the hydrogel [39]. The results show that dried RPAIG at 0.25 h significantly increases swelling and potentially improves the release of active compounds, while lyophilization produces very rapid swelling but mechanically weaker structures, the stability of which polyphenols can only partially compensate. Such comparative behavior of fresh and lyophilized RPAIG at pH 5.5 has not been described in the literature so far and provides new insights into the design of alginate-based carrier systems for the delivery of berry polyphenols.

### 2.6. Encapsulation Efficiency and Release Analysis of R. nigrum Alginate–Inulin Hydrogel

The encapsulation efficiency (*EE*%) of the developed hydrogel system was 91.6%, indicating that most of the extract was successfully retained within the hydrogel matrix. The high *EE*% demonstrates the effectiveness of the encapsulation process and suggests minimal loss of the extract during hydrogel formation. The elevated encapsulation efficiency can be attributed to the modified crosslinking procedure employed in this study. Instead of the conventional method of fully submerging the hydrogel formulation in the CaCl_2_ crosslinking solution, the hydrogel was formed by spraying the CaCl_2_ solution onto the formulation. This approach reduced direct contact between the extract-loaded hydrogel and the external crosslinking medium, thereby limiting the diffusion of the extract into the CaCl_2_ solution during gelation. Consequently, extract loss was minimized, resulting in a higher efficiency of 91.6%.

**Figure 9:**
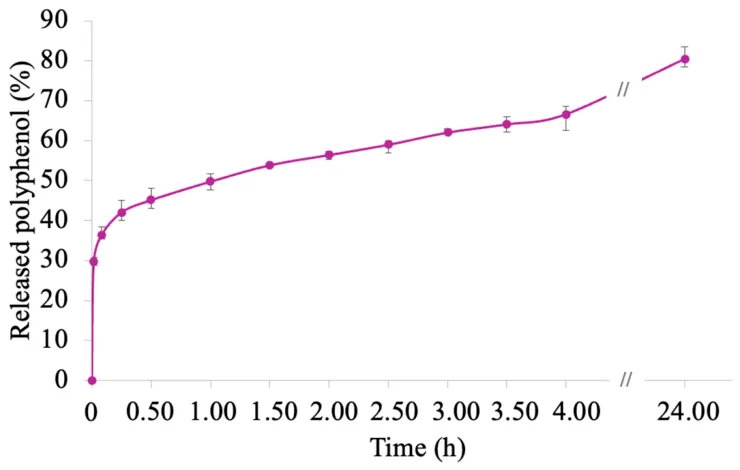
Polyphenol release from the RPAIG.

The release of polyphenols from the RPAIG followed a controlled two-stage release profile (Figure 9). A relatively high amount of polyphenols (30%) was detected within the first minute, which can be attributed to the rapid diffusion of surface-associated and weakly bound phenolic compounds upon initial hydrogel hydration. This initial release can be considered a beneficial burst effect, providing immediate availability of bioactive compounds, which is particularly advantageous in skin care applications where rapid antioxidant and anti-inflammatory action is required.

Following the initial phase, polyphenol release reached 80% within 24 h, and no further increase in release was observed after 48 h. This indicates that the release had reached a plateau after 24 h. This plateau may be attributed to polyphenol–matrix interactions. After the readily available fraction was released, the remaining polyphenols were likely retained more strongly within the matrix, limiting further release. Similar behavior has been reported for polyphenol-containing polymeric matrices and hydrogels [40]. The release profile was mostly linear, indicating diffusion-controlled transport through a structurally stable matrix. These features suggest that a fraction of polyphenols is reversibly associated with the polymer chains, leading to their more gradual release. The slight initial increase in the first 0.25 h reflects the characteristics of the “burst release” effect, where freely available polyphenols are released faster than those locked deeper in the matrix. The obtained results are consistent with the literature data on the release of polyphenols from alginate–inulin systems, where Fick-type diffusion and gradual hydrogel hydration dominate [41,42].

In this study, we used the spraying technique for crosslinking, since preliminary tests showed that full submergence of the hydrogel in the crosslinker led to loss of extract. This simple solution ensured an optimal encapsulation process and reduced leaching during crosslinking. The hydrogel provides predictable and controlled release of polyphenols, preserving the amount of biologically active compounds and demonstrating its potential in functional food products and delivery systems for biologically active substances [43].

The obtained results only reflect the comparative dynamics of the active substances released from the preparations under laboratory (in vitro) conditions. It is important to note that the model used in this work is a highly simplified version of diffusion and does not replicate the complex structure of human skin and its protective functions. However, simple pH response models are commonly used for preliminary studies.

### 2.7. Preliminary Assessment of Antibacterial Properties of Hydrogels

The antibacterial activity of RPAIG was compared with that of BLANKG, as shown in Table 2. Against *E. coli*, RPAIG reduced bacterial growth compared to BLANKG and the control (519 × 10^6^, 1310 × 10^6^, and 1560 × 10^6^ CFU, respectively), indicating an additional inhibitory effect associated with incorporation of RP96. In contrast, against *S. aureus*, RPAIG resulted in a higher bacterial count than BLANKG (94 × 10^6^ vs. 56 × 10^6^ CFU), indicating that RP96 incorporation did not enhance the antibacterial effect of the hydrogel matrix. This finding contrasts with the MIC profile of the free RP96 extract, which showed greater activity against *S. aureus* (MIC 3.1 mg/mL) than against *E. coli* (MIC 6.3 mg/mL). Therefore, the antibacterial behavior of RPAIG cannot be explained solely by diffusion limitations or by the concentration of released polyphenols relative to the MIC values of the free extract. The MIC assay and the hydrogel assay represent different experimental exposure systems: in the MIC assay, bacterial cells were directly exposed to the free extract, whereas in RPAIG, the extract constituents were incorporated into the hydrogel matrix and released gradually.

This discrepancy cannot be attributed to diffusion limitation alone, as incorporation into the hydrogel may alter the release and bioavailability of active compounds. Therefore, the MIC values obtained for the free extract cannot be directly extrapolated to the hydrogel system.

The complex mechanical and physical properties of the hydrogel, such as the swelling process, surface structure, pore size, and possible increase in viscosity in the release medium, might impact test results. It has been reported that bacteria can adhere and could be protected by the uneven surface of the hydrogel, thus distorting data [44]. Therefore, a defined conclusion on the antibacterial effect of hydrogels cannot be drawn from this antibacterial test alone.

Additionally, the behavior of bacteria in the medium and the hydrogel matrix should be considered. The size and motility of the bacteria could affect their survival and death rates in the experimental setting. Differing motility mechanisms between *E. coli* and *S. aureus* could contribute to the differences in results seen for free extract activity and hydrogel activity [45].

Therefore, future studies must include additional antibacterial assessment tests, and additional analysis of the released compounds and their antibacterial activity is required to explain the observed species-dependent response.

### 2.8. Current Limitations and Future Development of the Hydrogel System

The developed alginate–inulin hydrogel was successfully characterized, demonstrating its potential as a matrix for the topical delivery of *R. nigrum* pomace extract. The obtained results provide a basis for further development of natural composite hydrogels for topical applications. Similar potential has been reported for hydrogels incorporating grape pomace, supporting the use of plant-derived by-products as functional components in hydrogel-based delivery systems [46].

To further characterize the delivery behavior of the system, polyphenol release from the hydrogel was investigated under controlled in vitro conditions using phosphate buffer at pH 5.5. The release model provides useful information on the behavior of polyphenols within the hydrogel matrix and enables comparison under standardized conditions. However, these conditions do not reproduce the complex environment of human skin or wound tissue, where proteins, enzymes, cellular components, and dynamic pH changes could affect compound release and stability [46,47]. Therefore, the observed release profile cannot be directly predicted for skin penetration, bioavailability, or therapeutic efficacy. Similarly, the absence of clear self-healing behavior under the qualitative test conditions indicates that further investigation is required before this property can be considered a functional characteristic of the developed hydrogel.

Together, the present findings demonstrate the feasibility of developing an *R. nigrum* pomace extract-loaded alginate–inulin hydrogel, while also identifying formulation and biological factors that require further optimization. Future studies should investigate higher extract concentrations and optimize extract encapsulation efficiency and release kinetics. Additionally, the stability of released polyphenols is an important aspect to consider, as oxidation and degradation may influence their biological activity during storage and after release. Future studies could bring additional value by describing degradation products.

To determine the biological safety and potential wound-healing activity of the system, cytotoxicity studies and experiments using human fibroblasts are required. In addition, HPLC-MS/MS analysis would allow for more detailed characterization of the extracts’ bioactive constituents and help establish relationships between specific compounds and the observed biological effects. More physiologically relevant release models and comprehensive wound-healing studies would ultimately be required to determine the suitability of the developed hydrogel for wound-care applications.

## 3. Conclusions

This study highlights the valorization of *Ribes nigrum* pomace as a sustainable source of bioactive polyphenols for the development of alginate–inulin hydrogel systems. Compared with berry and juice extracts, *R. nigrum* pomace extract exhibited particularly high phenolic and tannin contents and demonstrated antibacterial potential.

The *R. nigrum* pomace extract was successfully incorporated into alginate–inulin hydrogels, forming structurally stable and amorphous matrices that allow for the gradual, diffusion-controlled release of polyphenols under skin-relevant conditions (pH 5.5). Incorporation of the extract affected the hydrogels’ physicochemical properties by increasing swelling capacity, improving viscoelastic recovery, decreasing viscosity, and improving *E. coli* inhibition. However, the antibacterial activity of the extract-loaded hydrogel remained limited under the tested conditions, indicating the need for further formulation optimization.

To the best of our knowledge, this work is the first report on directly integrating *R. nigrum* pomace extracts into an alginate–inulin hydrogel topical system, highlighting its potential as a sustainable, plant-based wound care application. These findings are an example of a circular bioeconomy—from food production waste to a potential medical biomaterial.

## 4. Materials and Methods

### 4.1. Study Area and Plant Material Collection and Preparation

The berries were collected in August 2024 from Aukspole village, Liksnas parish, Augsdaugava region, Latvia. Geographically, the site lies at 55°59′12″ N latitude and 26°24′8″ E longitude. The berries were labeled as blackcurrant, *Ribes nigrum* L., family: *Grossulariaceae*. After collection, they were washed, dried, and then frozen (−20 °C) for further analysis.

### 4.2. Materials and Reagents

All chemicals used in this study were of analytical grade and were used without any further purification unless otherwise stated. Purified water is hereafter referred to as water.

The reagents included UHPLC-grade methanol (Honeywell, CHROMASOLV, Seelze, Germany), potassium sodium tartrate tetrahydrate (Sigma-Aldrich, St. Louis, MO, USA), formic acid (Sigma-Aldrich, Darmstadt, Germany), gallic acid, ethanol, and Trolox (6-hydroxy-2,5,7,8-tetramethylchroman-2-carboxylic acid) (Acros Organics, Geel, Belgium), Folin–Ciocalteu reagent, phosphomolybdic tungstic reagent R, pyrogallol, sodium chloride, hide powder CRS, acetonitrile (Fisher Scientific, Loughborough, UK), sodium carbonate, sodium phosphate dibasic (Honeywell, Charlotte, NC, USA), inulin (Bioticana, Rendswühren, Germany), citric acid, calcium chloride hexahydrate (Carl Roth GmbH + Co. KG—Karlsruhe, Germany) and alginate (SpecialIngredients, Derbyshire, UK).

Reference standards for HPLC included chlorogenic acid (BLDpharm, Reinbek, Germany), caffeic acid, syringic acid, p-coumaric acid, ferulic acid, quercetin, rutin, kaempferol (Sigma-Aldrich, St. Louis, MO, USA), gallic acid, cyanidin-3-O-glucoside, cyanidin-3-O-galactoside, cyanidin-3-O-sambubioside, delphinidin-3-O-glucoside (PhytoLab, Vestenbergsgreuth, Germany), and delphinidin-3-O-galactoside (Extrasynthese, Genay, France).

The bacterial strains used in this study were *Escherichia coli* ATCC 35218 and *Staphylococcus aureus* ATCC 6538 (Microbiologics, St. Cloud, MN, USA). Mueller-Hinton broth (MHB, Oxoid Ltd., BasingstokeHampshire, UK) and Mueller–Hinton agar (Biomaxima, Lublin, Poland) were used for antibacterial testing.

### 4.3. Extract Preparation

**Figure 10:**
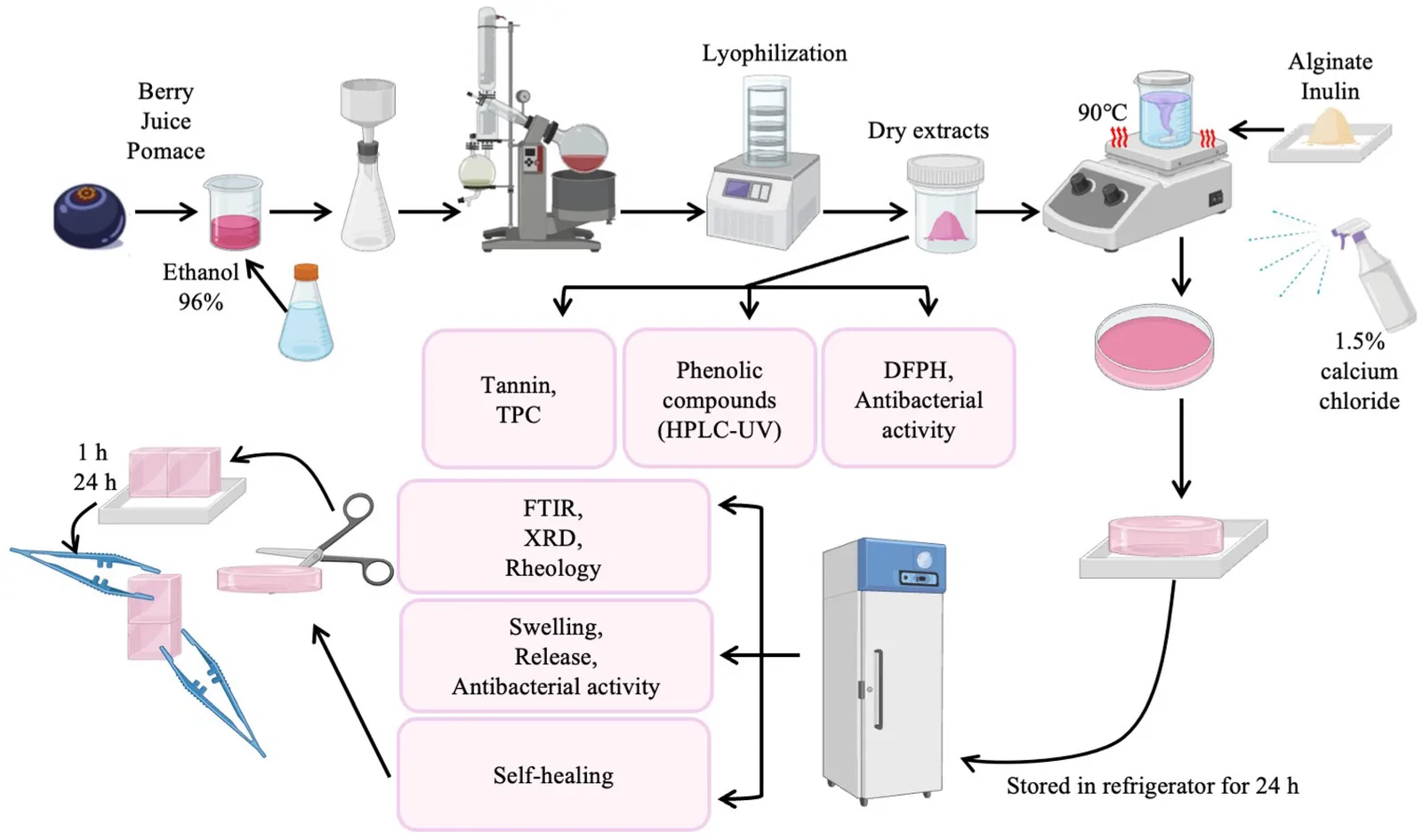
Development and characterization of *R. nigrum* extract and alginate–inulin hydrogel.

*R. nigrum* samples, including whole berries, berry juice, and berry pomace, were used for extract preparation. The code designations assigned to the obtained *R. nigrum* extracts: RJ96—juice extract; RB96—berry extract; RP96—pomace extract. Juice was obtained by mechanical pressing, and pomace was collected as a byproduct of juice production. The process is summarized in Figure 10.

Whole berries were crushed before solvent addition to improve contact between the plant material and the extraction solvent. The samples were mixed with a 96% ethanol solution at a 1:10 *v*/*w* ratio and left to macerate for 24 h at room temperature, as these conditions proved to be the most favorable in preliminary experiments (Antioxidant activity IC50 of RB in 50% ethanol was 6.2 mg/mL, RB in 70% was 5.5 mg/mL, and RB in 96% ethanol was 0.7 mg/mL). After maceration, the mixtures were filtered, the solvent was evaporated, and the obtained extracts were then freeze-dried for 24 h at −80 °C and 0.05 mbar.

### 4.4. Chemical Characterization of Extracts

#### 4.4.1. Determination of the Tannin Content in the Extracts

Tannin content was quantified using the protein precipitation method described in the European Pharmacopoeia, 8th edition [48], which allows for the estimation of condensed tannins in plant extracts.

Absorbance was measured at 760 nm using a UV/VIS spectrophotometer (Mettler-Toledo UV7, LabXTM, Greifensee, Switzerland), with water as the reference solution. The tannin content in dry extracts of *R. nigrum* was expressed as pyrogallol-equivalent percentages per dry extract. All measurements were performed in triplicate.

The tannin content was calculated according to the formula:

$$\mathrm{Tannins}\;=\frac{62.5\left(A_{1}-A_{2}\right)m_{2}}{A_{3}*\;m_{1}},$$

where *A*_1_ is the absorbance of total polyphenols, *A*_2_ the absorbance of non-adsorbed polyphenols, *A*_3_ the absorbance of the pyrogallol standard, *m*_1_ the mass of the extract sample (g), *m*_2_ the mass of pyrogallol (g), and 62.5 the conversion factor.

#### 4.4.2. Determination of the Total Polyphenol Content (TPC) in the Extract

TPC in *R. nigrum* extracts was determined using the Folin–Ciocalteu method with minor modifications, according to Mole GJ et al. [49]. A 0.20 mL sample of extract was mixed with 1.00 mL of 10% Folin–Ciocalteu reagent and 0.80 mL of sodium carbonate solution. After incubation in the dark for 30 min, absorbance was measured at 765 nm. Ethanol (0.20 mL) was used as the control. Results were expressed as gallic acid equivalents (mg GAE/g dry extract (DE)) using a gallic acid calibration curve (0.015–0.12 mg/mL, R^2^ = 0.9934) and calculated using the following formula:

$$C=a*y*\frac{V}{m}\;,$$

where ***C*** is the total polyphenol content expressed as mg GAE/g DE, ***a*** is the dilution factor, ***y*** is the concentration of the total phenolic compound according to the calibration curve in (mg/mL), ***V*** is the volume of ethanol (mL), and ***m*** is the mass of the dry extract (g).

#### 4.4.3. Determination of Phenolic Profiles by HPLC-UV Assay

The chromatographic analysis of the freeze-dried extracts was performed according to an already published HPLC-UV method by Bārzdiņa et al. [18]. The freeze-dried extracts were dissolved in 50% acetonitrile and filtered through a 0.45 µm syringe filter before analysis. A concentration of 10 mg/mL of all types of extract was used. The method was carried out on an UltiMate 3000 UHPLC+ focused system (Thermo Fisher Scientific, Waltham, MA, USA) using an Ascentis Express 90 Å AQ-C18 column (15 cm × 3.0 mm, 2.7 μm, Supelco, Darmstadt, Germany). Due to the characteristic absorption maxima of the polyphenols of interest, the detection wavelengths were set to 280 nm, 360 nm, and 520 nm. To identify the compounds of interest, the retention times of polyphenol standard solutions were compared to those of the extracts.

Peaks before 4 min and after 25 min were excluded from further analysis due to possible signals unrelated to the chemical composition of the extracts. Baseline correction and the creation of an average chromatogram for each type of sample at all three wavelengths were executed using SpectraGryph 1.2.17 software [50].

### 4.5. Characterization of the Biological Activities of Extracts

#### 4.5.1. Determination of Antioxidant Activity by DPPH Method

The antioxidant activity of the *R. nigrum* extracts was assessed using the DPPH (2,2-diphenyl-1-picrylhydrazyl) radical scavenging method, based on the guidelines for antioxidant assays for food components [51]. A 0.08 μg/mL DPPH solution was prepared in 96% ethanol, stored (at 4 °C) in the dark for 24 h and diluted until the working solution reached an absorbance of 1.0 ± 0.1 at 517 nm.

For the assay, various dilutions of the extracts were prepared. In each test tube, aliquots of the sample were mixed with distilled water and DPPH solution, vortexed, and then incubated in the dark at room temperature for 30 min. The decrease in absorbance was measured at 517 nm using a UV/Vis spectrophotometer (Mettler-Toledo UV7, LabXTM, Greifensee, Switzerland). Water with DPPH served as a control, while solvent blanks were used for baseline correction. Antioxidant activity was derived from IC50 calculations. The percentage of inhibition was calculated using the formula:

$$\frac{A_{\mathrm{control}}-A_{\mathrm{sample}}}{A_{\mathrm{control}}}\times 100\%,$$

where *A* is absorbance.

#### 4.5.2. Determination of the Antibacterial Activity of Extracts

Determination of minimum inhibitory concentrations (MIC) against Gram-negative (*E. coli* ATCC 35218) and Gram-positive (*S. aureus* ATCC 6538) bacteria was conducted according to EUCAST (European Committee on Antimicrobial Susceptibility Testing) [52]. The extracts were dissolved in distilled water (100 mg/mL) and stored at 4 °C until use. Antibacterial activity was assessed using a 96-well microdilution method in Mueller-Hinton Broth (MHB) [53]. Each well of the 96-well microplate (Sarstedt, Nümbrecht, Germany) was filled with 100 µL of MHB. Broth without added extract was used as a control. *R. nigrum* extract (100 µL) was serially diluted, ensuring equal final volumes. Finally, 100 µL of bacterial suspension was added to the wells. A bacterial inoculum of 5 × 10^5^ CFU/mL (100 µL) was used in this assay. All inoculations were performed under sterile conditions. After inoculation, the microplates were incubated for 24 h in a thermostatic incubator (Memmert IN 110, Schwabach, Germany) at 37 °C.

Following incubation, the wells were visually assessed, and absorbance at 600 nm was measured using a Tecan Infinite^®^ F50 microplate reader (Tecan, Männedorf, Switzerland).

The MIC was defined as the lowest concentration at which no visible bacterial growth was detected.

To determine the minimum bactericidal concentration (MBC), 10 µL from wells with concentrations one below and all above the MIC were plated on Mueller-Hinton agar plates and incubated for 24 h at 37 °C. Afterward, the plates were visually examined for bacterial growth.

### 4.6. Induction of Alginate–Inulin Hydrogels with R. nigrum Pomace Extract

Hydrogels were prepared using a thermal induction method [54], as shown in Figure 10. To prepare the blank alginate–inulin hydrogel, alginate (6% *w*/*v*) was dissolved in water under stirring at 90 °C at 500 rpm. As the viscosity increased, stirring continued manually with a glass rod. Then, inulin (80% *w*/*v*) was added, and the mixture was poured into silicone molds previously sprayed with a 1.5% calcium chloride solution.

For extract-containing hydrogels, RP96 dry extract (1% *w*/*v*) was added after inulin dissolution. The extract concentration was based on extract solubility in water and the hydrogel system, as well as previously obtained activity data.

Then, the mixture was transferred into silicone molds. All molds with hydrogels were sprayed with a 1.5% (*w*/*v*) calcium chloride solution from a distance of 15 cm and subsequently wrapped with cling film and stored at +4 °C for 24 h to allow the gel structure to develop. The silicone molds used for hydrogel preparation were 5 cm in diameter and 1 cm in height. Calcium chloride was applied by spraying to provide surface coverage; therefore, no fixed volume of calcium chloride solution or predefined stoichiometric alginate-to-calcium crosslinking ratio was used. The obtained hydrogels were assigned the following code designations: alginate–inulin hydrogel with *R. nigrum* pomace extract—RPAIG; alginate–inulin hydrogel—BLANKG.

### 4.7. Hydrogel Analysis

#### 4.7.1. Fourier Transform Infrared Spectroscopy with Attenuated Total Reflectance

Fourier transform infrared spectroscopy with attenuated total reflection (FTIR-ATR) was performed for the RP96 extract and freeze-dried hydrogel samples using a Thermo Fisher Scientific Nicolet iS5 (Thermo Fisher Scientific, Waltham, MA, USA) with a diamond ATR crystal. Spectra were recorded from 600 to 3800 cm^−1^ with 40 scans and a resolution of 4 cm^−1^, optical velocity of 0.4747, and an aperture of 100%, at room temperature. The results were processed using SpectraGryph 1.2.17 software [50].

#### 4.7.2. X-Ray Diffraction (XRD) Analysis

To assess the crystallinity of lyophilized hydrogel samples, XRD measurements were performed using a MiniFlex X-ray diffractometer (Rigaku Corporation, Tokyo, Japan) at 20 °C. Monochromatic CuKα radiation (λ = 1.6406 Å) was applied in a 2θ range of 10–60°. The instrument operated at 40 kV and 15 mA, with a scanning speed of 10°/min and a step size of 0.01°.

#### 4.7.3. Rheological Measurements

Rheological measurements were performed using an Anton Paar MCR 102e rheometer (Anton Paar GmbH, Graz, Austria) equipped with a PP50 parallel plate measuring system (Anton Paar GmbH, Graz, Austria) (plate diameter 50 mm). The initial gap was set to 1 N using automatic gap control. To determine the linear viscoelastic region (LVR), the onset of structural breakdown, and the flow point of the hydrogels, amplitude sweep (ASW) tests were conducted in oscillatory mode over a strain range of 0.01–1000% at a frequency of 1 Hz and a constant temperature of 37 °C. Structural recovery of the hydrogels after mechanical loading was evaluated using a three-interval thixotropy test (3ITT). The recovery test consisted of three cycles: two low-shear intervals (shear rate 0.1 s^−1^, 60 s) and one high-shear interval (5 s at 180 s^−1^). All rheological measurements were performed in triplicate. The obtained data were analyzed using Anton Paar RheoCompass^TM^ software (version 1.3).

#### 4.7.4. Qualitative Assessment of Macroscopic Self-Healing Test

To determine whether rheological recovery resulted in qualitative macroscopic self-healing, an automatic self-healing test without external stimuli was performed on RPAIG and on BLANKG based on the publication by Li Zhang, with minor modifications [55]. Similar self-healing assessment methods have also been employed in two other studies [56,57]. A general scheme of the test is shown in Figure 7. For the self-healing test, two pieces were cut from the hydrogel, then placed together and left in that position at room temperature (20–25 °C) for one hour and 24 h to evaluate both rapid and complete structural recovery. The hydrogels were stored in a closed Petri dish to maintain constant humidity and prevent drying. Then, hydrogel samples were tested by stretching with tweezers to qualitatively assess the mechanical integrity of the healed interface.

#### 4.7.5. Hydrogel Swelling Ratio

Hydrogel samples were prepared using two methods: air-drying at room temperature (20 °C) for 48 h, or freezing at −80 °C for 24 h followed by lyophilization.

The swelling behavior was evaluated according to the method described by Choong Jae Lee et al. [58], with minor modifications. The swelling degree of each hydrogel was calculated as the percentage increase in weight relative to the initial weight using the following formula:

*SR_w_* = (*W* − *W_o_*)/*W_o_* ∗ 100,

where *W*—the weight of the swollen hydrogel, *W_o_*—the initial weight of the hydrogel, and 100—the multiplier to express the result in %. Considering that the pH of healthy human skin typically ranges from 4.0 to 5.5, while acute wounds are neutral to slightly alkaline (pH 6.5–8.5) and the pH gradually decreases during the healing process [59,60,61], a phosphate–citric acid buffer with a pH of 5.5 was selected as the swelling developer. Swelling was performed for 24 h. The following time intervals were used in the test: 0, 0.25, 0.50, 0.75, 1.00, 1.25, 1.50, 1.75, 2.00 and 24.00 h.

#### 4.7.6. Encapsulation Efficiency and Polyphenol Release from the Hydrogel

The encapsulation efficiency (*EE*%) of the extract in the hydrogel was determined indirectly by measuring the amount of non-encapsulated extract remaining in the calcium chloride (CaCl_2_) crosslinking solution collected after encapsulation. Known absorbance values obtained using solutions of the extracts with known concentrations. Absorbance was measured at 515 nm. The absorbance of the collected CaCl_2_ solution served as a blank. The concentration of non-encapsulated extract was calculated from the known absorbance values. *EE*% was expressed as the percentage of the extract retained within the hydrogel according to the following equation:

*EE*% = *m*_1_/*m*_2_ × 100

where *m*_1_ is the initial mass of the extract used for hydrogel preparation and *m*_2_ is the mass of the extract detected in the collected CaCl_2_ solution after encapsulation.

For the release experiment, an RPAIG hydrogel (equivalent to 20 mg of extract, maintaining proportional composition) was placed in a cylinder. A magnetic stir bar was positioned centrally beneath the sample, and the system was stirred at 400 rpm. A total of 25 mL of phosphate acid buffer (pH 5.5) was used as the release medium. At each time point, 1 mL of sample was withdrawn and replaced with an equal volume of fresh buffer to maintain a constant total volume. The following time intervals were used in the test: 0.02, 0.08, 0.25, 0.50, 1.00, 1.50, 2.00, 2.50, 3.00, 3.50, 4.00 and 24.00, 48.00 h.

The release study was conducted at 37 °C using a MULTI-HS 6 DIGITAL Multiposition hotplate stirrer (VELP Scientifica, Usmate Velate, Italy) and continued for 24 h. Samples were collected from the center of the cylinder and centrifuged at 4000 rpm for 4 min using a SIGMA 1-14 centrifuge (Sigma Laborzentrifugen GmbH, Osterode am Harz, Germany). The amount of released polyphenols was determined using the technique described in Section 2.4.2. All measurements were performed in triplicate.

#### 4.7.7. Antibacterial Properties of Hydrogels

For the antibacterial testing of hydrogels, 3.5 g of BLANKG and RPAIG were placed in 24-well plates. MHB was added along with the bacterial suspension to make a final concentration of 5 × 10^5^ CFU/mL. The bacterial control contained broth together with bacteria, and the blank control contained broth. The plates were cultured at 37 °C for 24 h at 70 rpm (INFORS HT/Ecotron, Bottmingen, Switzerland). After 24 h, a serial dilution was prepared for each sample, and each was plated in triplicate on MHB agar to determine the number of CFU/mL [52].

## Figures and Tables

**Table 1:** Minimal inhibitory concentration (MIC), minimum bactericidal concentration (MBC) and MBC/MIC ratio of *R. nigrum* extracts against *E. coli* and *S. aureus*.

Sample	MIC (mg/mL)		MBC (mg/mL)		MBC/MIC Ratio	
	*E. coli*	*S. aureus*	*E. coli*	*S* *. aureus*	*E. coli*	*S. aureus*
RB96	6.3	12.5	25	25	4	2
RJ96	12.5	12.5	25	25	2	2
RP96	6.3	3.1	25	6.3	4	2

**Table 2:** Effect of alginate–inulin hydrogels on bacterial growth of *E. coli* and *S. aureus*.

Sample	*E. coli* (×10^6^ CFU)	*S. aureus* (×10^6^ CFU)
Initial concentration	33	33
Control (no hydrogel)	1560	220
BLANKG	1310	56
RPAIG	519	94

## Data Availability

The original data presented in this study are included in the article. Further inquiries can be directed to the corresponding author.
